# Collagenase treatment does not impair fiber contractile function in muscle biopsies from children with cerebral palsy

**DOI:** 10.14814/phy2.70645

**Published:** 2025-11-10

**Authors:** Faizan Syed, Latif Omerkhil, Venus Joumaa, Jason J. Howard, Timothy R. Leonard, Walter Herzog

**Affiliations:** ^1^ Human Performance Laboratory, Faculty of Kinesiology University of Calgary Calgary Alberta Canada; ^2^ Department of Orthopedic Surgery Nemours Children's Hospital Wilmington Delaware USA

**Keywords:** active properties, myofibrillar proteins, passive properties, passive stiffness, skinned muscle fibers

## Abstract

Cerebral palsy (CP) often presents with increased passive stiffness of the skeletal muscles, primarily due to increased collagen in the extracellular matrix. Collagenase from *Clostridium histolyticum* (CCH), an enzyme that degrades collagen, is used clinically to treat fibrotic conditions such as Dupuytren's contracture and Peyronie's disease. Although prior work demonstrated reduced passive stiffness in muscle bundles from children with CP following CCH incubation, its effects on active contractile properties remain unknown. Thus, this study was aimed at investigating the maximal active force, calcium sensitivity, and myofibrillar protein content (myosin and actin) after CCH incubation. Nine muscle biopsies from children with CP were used for skinned fiber mechanical testing at an average sarcomere length of 2.4 μm, and for myosin and actin analysis using 12% SDS‐PAGE. Maximal active stress (control 59.8 ± 24.2 kPa; CCH 63.2 ± 24.5 kPa; *p* = 0.51) and pCa₅₀ (control 6.04 ± 0.10; CCH 5.99 ± 0.18; *p* = 0.43) did not significantly differ. Similarly, the normalized myosin (control 1.000 ± 0.167, CCH 1.004 ± 0.178; *p* = 0.93) and actin (control 1.000 ± 0.336, CCH 1.107 ± 0.330; *p* = 0.231) content did not differ between conditions. These results suggest that collagenase does not impair the contractile function in muscle fibers from children with CP and thus might be a feasible treatment to reduce stiffness due to muscle fibrosis. Research into whole‐muscle force transmission following collagenase treatment is needed to evaluate its clinical viability.

## 1 INTRODUCTION

Cerebral palsy (CP) is a collection of motor disorders arising from an early‐childhood non‐progressive brain injury (Vitrikas et al., 2020). Affecting approximately 2 per 1000 live births, CP remains the most common cause of childhood physical disability (Oskoui et al., 2013). Clinically, children with CP present with spasticity, impaired muscle size and function, and increased muscle tone and passive stiffness (e.g., Armand et al., 2016; Gage & Novacheck, 2001; Graham, 2005; Graham et al., 2016; Graham & Selber, 2003; Howard & Herzog, 2021; Sutherland, 1978; Sutherland & Davids, 1993). It has been suggested that this elevated inherent muscle stiffness results from overstretched sarcomeres (Leonard et al., 2019; Lieber & Fridén, 2002, 2019; Smith et al., 2011) and increased collagen content within the extracellular matrix (ECM; Booth et al., 2001; Smith et al., 2011).

Currently, there are no clinical procedures specifically aimed at directly reducing collagen‐associated passive stiffness in children with CP. However, interventions such as surgical tendon release or intramuscular botulinum toxin type A (BoNT‐A) injections into affected muscles are used to restore the range of motion in targeted joints (Howard et al., 2023). Although BoNT‐A injections effectively reduce muscle contractures temporarily, these injections frequently cause transient pain and weakness (Lukban et al., 2009). Moreover, repeated BoNT‐A injections induce fat infiltration and fibrosis in both target and adjacent muscles (Fortuna et al., 2011, 2015), potentially exacerbating existing muscle weakness and stiffness. Surgical interventions, such as tendon release and hamstring lengthening, are effective but remain invasive and risk recurring contractures (Beals, 2001). Thus, there remains a critical need for an accessible, safe, and non‐invasive treatment capable of decreasing CP‐associated muscle stiffness without causing debilitating side effects.

Collagenase, an enzyme that degrades collagen, the major component of the ECM, has been approved by the U.S. Food and Drug Administration (FDA) to treat fibrotic diseases such as Dupuytren's and Peyronie's (Egui Rojo et al., 2014; Honig, 2014; Hurst et al., 2009). Collagenase injections effectively eliminate contractures and increase motion in patients with advanced Dupuytren's disease without causing permanent side effects, as demonstrated in placebo‐controlled, randomized, double‐blind trials (Hurst et al., 2009). Similar safety and efficacy of collagenase was observed in trials evaluating collagenase injections for Peyronie's disease (Egui Rojo et al., 2014; Honig, 2014). Despite its success in treating fibrotic diseases, the potential of collagenase to specifically reduce CP‐associated muscle stiffness has not yet been systematically investigated.

In a previous study, we treated hip adductor muscle fiber bundles from children with CP with a range of collagenase *Clostridium histolyticum* (CCH) doses and demonstrated a linear dose‐dependent response between CCH concentration and collagen depletion (Howard et al., 2023). We then investigated whether collagen degradation using CCH was effective in reducing the passive stiffness of intact muscle fiber bundles from children with CP. Using a CCH concentration of 350 U/mL, there was an approximately 51% reduction in Young's modulus and a significant drop in both peak and steady‐state passive stresses obtained following a series of stress‐relaxation tests at controlled bundle lengths (*p* < 0.005), without altering total protein content. However, that proof of concept study focused purely on the passive mechanics of intact muscle fiber bundles and did not examine whether CCH had negative effects on the contractile function or the content of proteins that are known to affect muscle contraction and force capacity.

Thus, to fill this gap, this study was aimed at investigating the effects of CCH on muscle biopsies from children with CP by specifically evaluating the active and passive properties of single skinned muscle fibers and myosin and actin content after CCH treatment. Because skinning substantially removes the ECM, passive stiffness in these skinned single fibers is primarily titin‐based. Therefore, unlike the reduction in passive stiffness observed in the intact collagen‐rich bundles, we hypothesized that active and passive force, calcium sensitivity, and contractile protein content would remain unaffected in single muscle fibers following incubation in CCH.

## 2 MATERIALS AND METHODS

### 2.1 Subjects

Nine biopsies of the semitendinosus and semimembranosus muscles were obtained from children (ages 6–16 years, 5 males, 4 females) with severe CP (Gross Motor Function Classification System [GMFCS] levels IV and V) undergoing hip adductor tendon release to correct progressive hip subluxation or dislocation. Patients were excluded if they had previous hip adductor surgery, a non‐spastic motor type, lacked adductor contractures, or had neuromuscular conditions other than CP. Muscle samples were collected surgically, immediately flash‐frozen in liquid nitrogen, and stored at −80°C until analysis. Ethics approval and written informed consent from patients and/or their guardians were obtained prior to the project (University of Calgary: REB19‐1823_REN4, Nemours Children's Hospital: 687629). All human‐participant related activities were conducted in accordance with the Declaration of Helsinki.

### 2.2 Skinned muscle fiber preparation

Frozen biopsies stored at −80°C were first incubated overnight in a rigor/glycerol solution (50:50 v/v) at −20°C and then chemically skinned in a relaxing solution containing 1% Triton X‐100 for 3 h on ice at 4°C (Feng & Jin, 2020; Joumaa et al., 2025; Ma et al., 2022). This freezing and then skinning protocol has been shown to preserve the active and passive properties of skeletal and cardiac muscle samples (Joumaa et al., 2025; Ma et al., 2023; Milburn et al., 2022). After thorough washing in relaxing solution, each sample was pinned in a Petri dish containing either CCH (350 U/mL in relaxing solution; CCH lyophilized powder, Worthington Biochemical Corp., Lakewood, NJ, USA, Cat. No. LK003240) or relaxing solution alone (control) (Howard et al., 2023). Dishes were then incubated at 37°C for 45 min in a water bath, followed by several washes in relaxing solution containing protease inhibitors (Complete, Roche Diagnostics, Quebec, Canada, Cat. No. 11836145001, 1 tablet per 50 mL of solution) to stop CCH activity. Samples were stored in a rigor/glycerol solution at −20°C and used to isolate and test single fibers within 2 days.

Using a binocular microscope (Nikon SMZ1500, Tokyo, Japan), individual fibers were dissected and transferred to an experimental glass chamber (Model 308, Aurora Scientific Inc., Ontario, Canada) filled with relaxing solution. One end of each fiber was glued to a length controller (Model 322C) and the other to a force transducer (Model 400A; Aurora Scientific Inc.), enabling precise length adjustments and force recordings. Average sarcomere length (SL) was measured by *He‐Ne* laser diffraction (Edman & Flitney, 1982) in relaxing solution immediately before activation. Fibers were adjusted with a length controller to a target average SL of 2.4 μm. Fiber diameter was then measured using a binocular microscope to calculate cross‐sectional area (assuming a cylindrical geometry). Recorded fiber forces were converted to stress by normalizing to the cross‐sectional area. From each biopsy, two to seven fibers were tested, yielding a total of 31 CCH‐treated and 28 control fibers. Fibers from the same CP biopsy and treatment were grouped together, yielding one median value per CP sample and treatment (9 CCH and 9 control in total). All measurements were conducted at room temperature (~22°C).

### 2.3 Mechanical tests

#### 2.3.1 Maximal active stress

Skinned fibers were positioned at an average SL of 2.4 μm in relaxing solution, then activated by moving the fiber to a washing solution and then a high‐calcium activating solution (pCa = −log_10_[Ca^2+^] = 4.2, Figure S1). After a plateau was achieved, fibers were returned to relaxing solution. The peak force achieved during activation was recorded and normalized by cross‐sectional area to yield maximal active stress.

#### 2.3.2 Passive stress

Fibers were set at an average SL of 2.4 μm, stretched passively in 2 s to a final SL of 2.6 μm and held for 40 s to allow for stress relaxation (Figure S2). This test was repeated for stretches to final SLs of 2.8, 3.0, 3.2, and 3.4 μm (Leonard et al., 2019). Passive force was defined as the steady‐state force at the end of each 40 s hold and normalized by cross‐sectional area to calculate passive stress.

#### 2.3.3 Calcium sensitivity

Fibers were set to an average SL of 2.4 μm and subsequently exposed to activating solutions at pCa values of 7.0, 6.8, 6.6, 6.4, 6.2, 6.0, 5.8, 5.4, and 4.2, corresponding to ascending calcium concentrations (Moss, 1979). At each pCa step, the fiber was incubated until force reached a steady plateau (approximately 1 min), and that force was recorded (Figure S3). Force–pCa data were fitted to the Hill equation using SigmaPlot software (SigmaPlot version 16.0 for Windows, SigmaPlot Software, Palo Alto, California USA) to derive the Hill slope and the pCa₅₀ (the pCa producing half‐maximal force serving as a measure of calcium sensitivity).

### 2.4 Protein analysis

#### 2.4.1 Myofibrillar protein content

Muscle biopsies from children with CP were incubated at 37°C for 45 min in either CCH (350 U/mL) or relaxing solution. After incubation, samples were thoroughly rinsed in relaxing solution, gently blotted dry, and weighed. Each sample was then placed in a protein‐extraction buffer (50 mM Tris, 500 mM NaCl, 20 mM Na₄P₂O₇, 1 mM EDTA, 1 mM DTT, and 1 tablet of protease inhibitors (Complete, Roche Diagnostics, Quebec, Canada) per 50 mL of solution, pH = 7.4), homogenized for 15–25 s, vortexed, sonicated for 15 min, and centrifuged at 13000 rpm for 15 min at 4°C. Protein content in the supernatant was quantified (Smith et al., 1985) via bicinchoninic acid (BCA) assay (Pierce, ThermoFisher Scientific, Illinois, USA, Cat. No. 23227). Finally, the extracts were adjusted to a final concentration of 3 mg of protein/mL in Laemmli solubilization buffer (62.5 mM Tris–HCl, 10% glycerol, 2% SDS, 5% β‐mercaptoethanol, 0.02% bromophenol blue, and protease inhibitors, pH = 6.8) for SDS–PAGE.

#### 2.4.2 Myosin heavy chain and actin content

Myosin heavy chain (MHC) and actin content were evaluated using 12% SDS–PAGE. Solubilized myofibrillar extracts (1.5 μL per well, volume chosen based on calibration gel to avoid saturation; Figure S4) were separated in 0.75 mm–thick gels using a Bio‐Rad (USA) Mini‐PROTEAN Tetra system at 130 V for 2 h at room temperature. Each gel was loaded with an actin standard (Sigma‐Aldrich, Missouri, USA, Cat. No. A2522), and a supporting gel was run with a molecular weight ladder (Figures S5 and S6). Gels were then stained with Coomassie Brilliant Blue for 1 h and destained immediately in 50% ethanol/7% acetic acid for 10 min, followed by 5% ethanol/7% acetic acid for 1 h. After destaining, gels were scanned on a Bio‐Rad GS‐800 scanner, and band optical densities were quantified with ImageJ (National Institutes of Health, Bethesda, MD, USA). To represent the proteins individually, actin and myosin optical densities were normalized to the control group mean for each protein. Each sample was done in two replicates, from which the median values were calculated to represent each subject.

### 2.5 Solutions

Relaxing solution (in mM): potassium propionate (170), magnesium acetate (2.5), MOPS (20), K2EGTA (5), and ATP (2.5), at a pH of 7.0. For every 100 mL of relaxing solution, 1 tablet of protease inhibitors was added. Washing solution (in mM): potassium propionate (185), magnesium acetate (2.5), MOPS (10) and ATP (2.5), at a pH of 7.0. Activating solution (in mM): potassium propionate (170), magnesium acetate (2.5), MOPS (10), ATP (2.5) and free Ca^2+^ buffered with EGTA (CaEGTA and K2EGTA mixed to obtain a pCa 4.2 value), at a pH of 7.0.

### 2.6 Statistical analysis

Two‐tailed paired *t*‐tests (or Wilcoxon signed‐rank test for passive stress results) were used to compare the outcome measures of maximal active stress, pCa_50_, Hill slope, myosin, and actin content between the CCH and control groups. Normality was assessed using Shapiro–Wilk, and residual Q‐Q plots with a significance set at *α* = 0.05. The statistical analyses were performed using GraphPad (GraphPad Prism version 10.0.0 for Windows, GraphPad Software, Boston, Massachusetts USA) and the level of significance was set at *α* = 0.05. Results are shown as means ± SD.

## 3 RESULTS

### 3.1 Mechanical tests

#### 3.1.1 Maximal active stress

**FIGURE 1:**
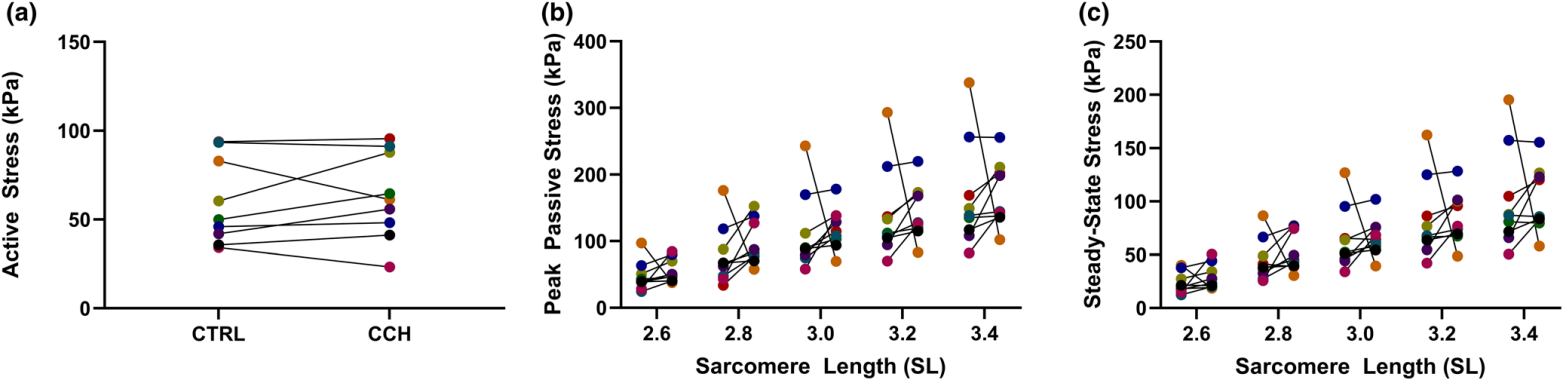
Effect of collagenase *Clostridium histolyticum* (*CCH*) on active and passive contractile properties of skinned muscle fibers from children with cerebral palsy compared to control (CTRL). For each figure (*n* = 9, each subject is the median of 2–7 replicates), results of individual subjects are shown, with data from the same subject in the CTRL and CCH conditions connected with a black line. (a) Maximal active stress at a sarcomere length (SL) of 2.4 μm in CTRL (Mean ± SD, 59.8 ± 24.2 kPa) and CCH (63.2 ± 24.5 kPa) treated fibers (*p* = 0.51, paired *t*‐test). (b) Peak passive stress at SLs 2.6–3.4 μm in CTRL (left) and CCH (right) treated fibers (*p* ≥ 0.16, Wilcoxon signed‐rank test). (c) Steady‐state passive stress at SLs 2.6–3.4 μm in CTRL (left) and CCH (right) treated fibers (*p* ≥ 0.098, Wilcoxon signed‐rank test).

CCH treatment did not significantly impact maximal active stress at an average SL of 2.4 μm. The mean active stresses were 59.8 ± 24.2 kPa for the control and 63.2 ± 24.5 kPa for the CCH group (95% CI [−7.8, 14.5]; paired *t*(8) = 0.70, *p* = 0.51) (Figure 1a).

#### 3.1.2 Passive stress

Peak and steady‐state passive stresses were not significantly different between control and CCH groups at all sarcomere lengths from 2.6 to 3.4 μm (peak stress: *p* ≥ 0.16; steady‐state stress: *p* ≥ 0.098) (Figure 1b,c).

#### 3.1.3 Calcium sensitivity

**FIGURE 2:**
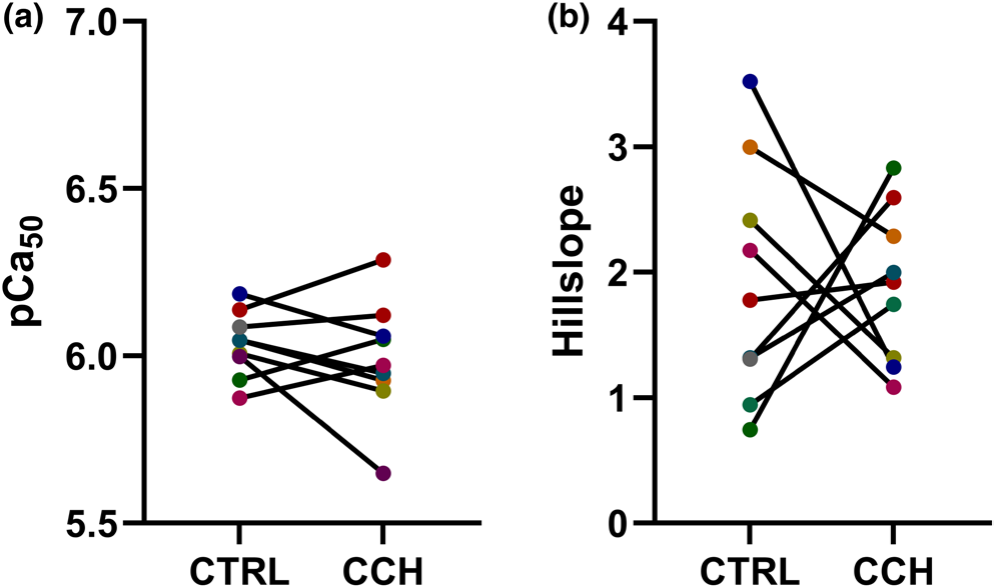
Effect of collagenase *Clostridium histolyticum* (*CCH*) on calcium sensitivity (pCa_50_) and Hill slope in skinned muscle fibers from children with cerebral palsy compared to control (CTRL). For each figure (*n* = 9, each subject is the median of 2–7 replicates), results of individual subjects are shown, with data from the same subject in the CTRL and CCH conditions connected with a black line. (a) pCa_50_ (−log_10_ [Ca^2+^] required to produce 50% of maximal force) of CTRL (Mean ± SD, 6.04 ± 0.10) and CCH (5.99 ± 0.18) treated fibers (*p* = 0.43, paired *t*‐test). (b) Hill slope of (CTRL (Mean ± SD, 1.91 ± 0.94) and CCH (1.89 ± 0.61) *p* = 0.97, paired *t*‐test) treated fibers.

Force‐pCa curves for both control and CCH‐treated groups exhibited typical sigmoidal profiles. CCH Treatment did not significantly impact pCa_50_ and the Hill slopes between groups. The mean pCa_50_ values were 6.04 ± 0.10 in control versus 5.99 ± 0.18 with CCH treatment (95% CI [−0.080, 0.17]; paired *t* (8) = 0.84, *p* = 0.43, Figure 2a). The mean Hill slopes were 1.91 ± 0.94 in control, and 1.89 ± 0.61 with CCH incubation (95% CI [−1.04, 1.10]; paired *t* (8) = 0.042, *p* = 0.97, Figure 2b). Because fibers were maximally activated twice (during the maximal active stress test and again at the end of the force–pCa protocol), the forces were compared between both time points as a quality check. The final activation was, on average, 6.9% lower (mean difference of 9.65 kPa), indicating minimal loss.

### 3.2 Protein analysis

#### 3.2.1 Myosin

**FIGURE 3:**
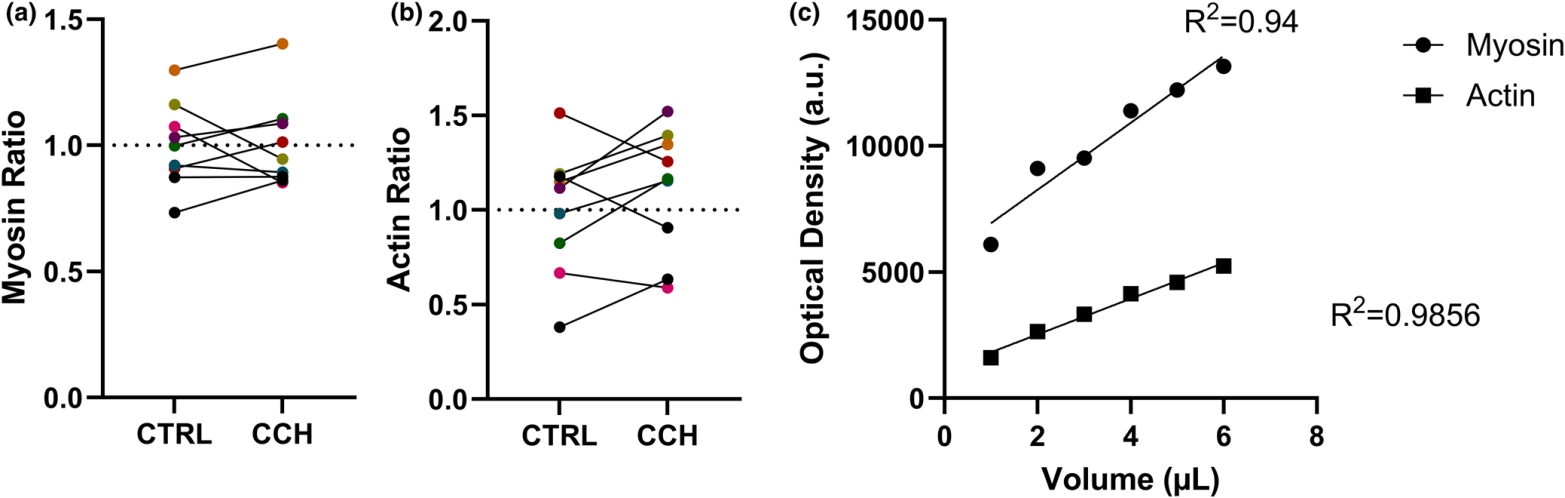
Effect of collagenase *Clostridium histolyticum* (*CCH*) on actin and myosin content in muscle biopsies from children with cerebral palsy compared to control (CTRL). For each figure (*n* = 9, each subject is the median of duplicate lanes), results of individual subjects are shown, with data from the same subject in the CTRL and CCH conditions connected with a black line. (a) Myosin, expressed relative to the control mean (CTRL = 1.00; dotted line, CTRL = 1.00 ± 0.167, CCH = 1.00 ± 0.178, *p* = 0.934). (b) Actin, expressed relative to the control mean (CTRL = 1.000 ± 0.336, CCH = 1.107 ± 0.330, *p* = 0.231). (c) A 12% SDS‐PAGE calibration gel (Figure S4) was used to evaluate the relationship between the amount of protein loaded per well and the resulting optical density using ImageJ.

Myosin content normalized to the control mean was not significantly different between control and CCH (CTRL 1.00 ± 0.167, CCH 1.00 ± 0.178; 95% CI [−0.101, 0.109]; paired *t*(8) = 0.085, *p* = 0.934; Figure 3a).

#### 3.2.2 Actin

Similarly, actin content normalized to the control mean was also not significantly different between the control and CCH treatments (CTRL 1.000 ± 0.336, CCH 1.107 ± 0.330; 95% CI [−0.0838, 0.299]; paired *t*(8) = 1.296, *p* = 0.231; Figure 3b). A calibration plot showing the relationship between sample volume loaded and optical density was used to avoid saturation (Figure 3c).

## 4 DISCUSSION

In our previous work, we demonstrated that incubation with CCH reduced collagen‐based passive stiffness in intact muscle fiber bundles from children with CP, without affecting total protein content (Howard et al., 2023). Building on this, the current study was aimed at determining whether CCH affects active and passive mechanical properties at the sarcomere level. Mechanical characteristics including maximal active stress at a SL of 2.4 μm, passive stress at SLs of 2.6–3.4 μm, calcium sensitivity (pCa_50_), and Hill slope were evaluated in skinned single fibers. Additionally, myosin and actin contents were quantified through optical density measurements via SDS‐PAGE. Our results indicated no significant differences between CCH‐treated and control groups across all measured parameters, confirming that CCH does not impair muscle fiber mechanical properties at the cellular level.

### 4.1 CCH and fiber mechanical properties

Active force in skinned muscle fibers relies on the cycling of cross‐bridges formed between the thin (actin) and thick (myosin) filaments (Gordon et al., 1966; Huxley, 1957). Given that these proteins are not substrates for CCH, the observed lack of significant difference in maximal active stress between groups was anticipated. To further confirm contractile function post‐CCH treatment, myosin and actin content were assessed using SDS‐PAGE. Degradation would suggest protein cleavage; however, no significant changes in actin, and myosin content were observed, reinforcing that the sarcomeric myofilament proteins were preserved. Another myofilament protein, titin, was not tested using SDS‐PAGE due to its susceptibility to degradation in response to heat. Instead, passive stretching of the muscle fibers at various sarcomere lengths was performed to determine titin elasticity. In single skinned fiber and myofibril preparations, titin is the primary and virtually exclusive contributor to passive force (Bartoo et al., 1997; Granzier et al., 1997; Linke et al., 1994). At all SLs there was no significant difference in passive stress between the CCH and control groups, indicating CCH did not compromise titin function.

Furthermore, calcium sensitivity testing was conducted to evaluate potential indirect damage to the regulatory proteins, troponin and tropomyosin. Calcium sensitivity, reflected by pCa_50_, denotes the efficiency of troponin and tropomyosin regulatory proteins in facilitating cross‐bridge formation at given Ca^2+^ concentrations (Palmer & Kentish, 1994; Ruff, 1989). The Hill slope measures the cooperative response of fibers to calcium binding, with decreased values typically observed in damaged or diseased muscle fibers (Mokbel et al., 2013). Disorders such as nemaline myopathy have shown significantly reduced pCa_50_ and Hill slopes compared to controls (de Winter & Ottenheijm, 2017; Mokbel et al., 2013). Our results found no significant differences in calcium sensitivity metrics between CCH‐treated and control fibers, further supporting the conclusion that CCH does not damage contractile proteins.

It is important to note that the mean maximal active stress produced by both CCH and control group fibers was approximately 60–63 kPa. Studies have shown single fiber stress to vary from 106 to 227 kPa in healthy vastus lateralis muscle skinned fibers, and approximately 79 kPa in muscle fibers isolated from adductor longus muscles of children with CP (Choi & Widrick, 2010; Joumaa et al., 2025). One explanation for the differing stress values is the incubation at 37°C for 45 min. Skinned muscle fibers have been noted to be stable up to 35°C, with subsequent heating causing damage and diminished calcium sensitivity (Stephenson & Williams, 1985). However, the values obtained for calcium sensitivity (pCa_50_) were within ranges previously reported for both groups (pCa_50_: 5.83–5.96; Hill slope: 1.74–4.94; Teigen et al., 2020; Bottinelli & Reggiani, 2000). Additionally, the passive stress values were also similar to previously reported values from muscle fibers from children with CP (15–100 kPa, 2.4–3.6 μm; Joumaa et al., 2025). Thus, our results indicate that the mechanical properties of the skinned fibers tested in this study were not compromised compared to previous literature. An additional explanation for the low active stress in our fibers is that the intrinsic contractile capability in CP fibers may be inherently diminished due to underlying disease pathology. Joumaa et al. (2025) showed that maximal active stress produced by skinned single fibers from the adductor longus of children with CP was 79 kPa compared to 170 kPa from healthy vastus lateralis fibers. Similar values have been reported in other muscle impairment diseases such as Duchenne muscular dystrophy, and nemaline myopathy (Fink et al., 1990; Horowits et al., 1990; Joumaa et al., 2025; Lawlor et al., 2011). Similarly, a pilot study performed to establish the experimental protocol for our study tested 10 control rabbit psoas skinned fibers. The mean maximal active stress at 2.4 μm in these fibers was 119.0 kPa (results not shown), similar to values previously reported for rabbit psoas skinned fibers tested at 15°C–22°C (Joumaa & Herzog, 2014). These fibers underwent identical heating treatment as the fibers isolated from CP muscle indicating that the inherent contractile ability of the muscle largely determines the fiber maximal active stress. Furthermore, it is possible that collagenase treatment alters SL in the muscle biopsies. However, SLs were adjusted to 2.4 μm after treatment and immediately before activation, suggesting active stress values in our CCH fibers are not reduced due to changes in SL following collagenase treatment. Therefore, we conclude that incubation at 37°C was a valid method for replicating physiological conditions without damaging the contractile function of the muscle fibers.

### 4.2 CCH to alleviate passive stiffness in CP muscles

Given collagen's crucial role in preserving muscle structural integrity and in force transmission at the fascicular and whole‐muscle levels, excessive collagen reduction or irregularities in the network could compromise muscle structure and force transmission to the tendons in vivo (Finni et al., 2023; Gillies & Lieber, 2011; Huijing, 1999; Provenzano & Vanderby, 2006; Purslow, 2010). Specifically, abundant CCH would decrease the collagen content in the endomysium, perimysium or epimysium making the connective tissue network compliant and the muscle fibers lose their organizational framework, thereby decreasing the maximal active force output (Maas & Sandercock, 2010). However, because the ECM content is significantly increased in muscles from children with CP (Smith et al., 2011), appropriate CCH doses could normalize collagen to physiologically optimal levels without compromising the muscle structural integrity and force transmission. However, to date, systematic studies on intramuscular CCH dosing are lacking.

A future objective for this project is to investigate the effect of CCH on whole muscle structural integrity and force transmission in an animal model of CP. Unfortunately, to date, there is no reliable animal model for CP. Therefore, muscles from mdx mice, a model for Duchenne muscular dystrophy which, similar to muscles from children with CP, exhibit drastic collagen accumulation (Giovarelli et al., 2022; Huebner et al., 2008; Smith & Barton, 2014), could be used to investigate the in vivo efficiency of CCH in treating excessive collagen and increased passive stiffness. Furthermore, it is imperative to understand in animal models how CCH affects muscle surrounding tissues and blood vessels, and whether CCH injected into a target spastic muscle can diffuse to the blood stream and circulate to vital organs. Determining optimal CCH doses to safely reduce stiffness without compromising force transmission, blood vessels and vital organs could support the clinical application of CCH in managing CP‐associated stiffness or even mitigating adverse effects from common clinical interventions like BoNT‐A injections.

BoNT‐A injections are widely prevalent in the treatment of spasticity. Functionally, it restricts the transmission of acetylcholine and thereby downstream muscle activation (Aoki, 2004; Aoki & Guyer, 2001; Dolly & Aoki, 2006; Gracies, 2004; Howard et al., 2023; Meunier et al., 2002; Yaraskavitch et al., 2008). However, it has been known to cause significant muscle weakness and fibrosis even in non‐target muscles (Ansved et al., 1997; Bakheit, 2006; Fortuna et al., 2015; Phadke et al., 2016). Although CCH does not address spasticity, its targeted collagen reduction may restore the range of motion and facilitate physical therapy participation, potentially offsetting muscle weakness.

### 4.3 Limitations

Collagen and the ECM are important for the modulation of excitation–contraction coupling (ECC). For example, knockout of collagen VI, an abundant muscle ECM protein, results in sarcoplasmic reticulum (SR) dysfunction in animal models, highlighting the influence of collagen on ECC (Bernardi & Bonaldo, 2008). In cardiac muscle, supplementation of cultured cardiomyocytes with collagen type I modulates SR Ca^2+^ handling, further supporting a relationship between collagen and ECC (Lu et al., 2011). It has also been shown that calcium release from the SR in intact muscle fibers isolated with type I collagenase remains unchanged; yet mitochondrial calcium control is impaired (Gineste et al., 2022). This indicates that collagenase‐related ECM disruption may affect various components of ECC that our permeabilized single‐fiber protocol cannot necessarily capture. Therefore, caution is warranted when translating findings from skinned fibers incubated in collagenase to in vivo muscle.

Fiber typing is often performed alongside single‐fiber mechanical testing; however, fiber typing was not feasible in this study due to tissue‐handling constraints. The notably thin and short fibers had insufficient material to yield interpretable SDS–PAGE results. To minimize potential differences in fiber types, CCH and control fibers were sampled from adjacent regions of each biopsy, and multiple fibers were tested per biopsy to better represent each participant. This is a limitation and will be addressed in future work.

Glycerol storage can increase passive stiffness particularly at extended sarcomere lengths, while active properties remain unaffected (Han et al., 2025). Because both CCH‐treated and control fibers were stored identically and analyzed within participants, relative treatment comparisons are unlikely to be biased, but absolute passive values may be shifted upward compared to fibers that are not incubated in glycerol.

## 5 CONCLUSION

From the results of this study, we conclude that CCH does not impair the contractile function in muscle fibers from children with CP. These findings provide a critical first step in establishing the safety of intramuscular CCH injections to reduce CP‐associated inherent muscle stiffness, thereby improving range of motion without compromising muscle fiber contractile function. Additional research into whole‐muscle interactions and force transmission following CCH treatment remains necessary to evaluate its clinical viability.

## AUTHOR CONTRIBUTIONS

**Faizan Syed**, **Latif Omerkhil**, **Venus Joumaa**, **Jason J. Howard**, **Timothy R. Leonard**, and **Walter Herzog**: Conception and design of research, Interpreted results of experiments, Drafted manuscript, Edited and revised the manuscript, and Approved final version of the manuscript. **Faizan Syed**, **Latif Omerkhil**, **Venus Joumaa**: Analyzed data and Performed experiments. **Faizan Syed**, **Latif Omerkhil**, **Venus Joumaa**, **Walter Herzog**: Prepared figures.

## FUNDING INFORMATION

This research was financially supported by the Natural Sciences and Engineering Research Council of Canada (NSERC, RGPIN‐2020‐03920), the Canadian Institutes of Health Research (CIHR, PJT‐178168), and Dr. Benno Nigg Chair in Biomechanics, Mobility and Longevity (RT751928).

## CONFLICT OF INTEREST STATEMENT

The authors declare no conflicts of interest.

## Supporting information


**Figure S1.** Maximal active force trace at an average sarcomere length (SL) of 2.4 μm (diameter of 0.054 mm). A single skinned muscle fiber was set to SL = 2.4 μm in relaxing solution, transferred through a washing solution, and then to an activating solution (pCa 4.2) to maximally activate the fiber. Sudden changes in the force trace correspond to the testing system transferring between baths.

**Figure S2.** Passive staircase force trace across increasing sarcomere lengths (SL 2.6–3.4 μm, diameter: 0.083 mm). Peak force was collected at the highest point during the stretch, and steady‐state force was collected at the plateau before the next stretch began.

**Figure S3.** Calcium‐sensitivity force trace at an average sarcomere length (SL) of 2.4 μm with a diameter of 0.033 mm. A single skinned muscle fiber was exposed to solutions of increasing [Ca^2+^] (pCa 6.8, 6.6, 6.4, 6.2, 6.0, 5.8, 5.6, 5.4). The sudden changes in force are due to the switching of pCa solutions. Baseline force was calculated and subtracted from the active force, which was collected at the plateau before pCa solution change.

**Figure S4.** A 12% SDS–PAGE gel was loaded with increasing volumes of the same muscle sample extract to demonstrate the linear range of optical density for densitometry of myosin and actin.

**Figure S5.** SDS–PAGE gel of collagenase‐incubated samples. The last lane contains a purified actin standard used for band identification.

**Figure S6.** SDS‐PAGE gel of samples run alongside a molecular weight ladder with myosin and actin labeled. Alignment with the ladder was used to verify band identity.

## Data Availability

The data for this study are available on request from the corresponding author.
