# Supplementary figures and images for: Collagenase treatment does not impair fiber contractile function in muscle biopsies from children with cerebral palsy
Source: Physiol Rep. 2025 Nov 10;13(21):e70645. doi: 10.14814/phy2.70645 (PMC12602256; doi:10.14814/phy2.70645)

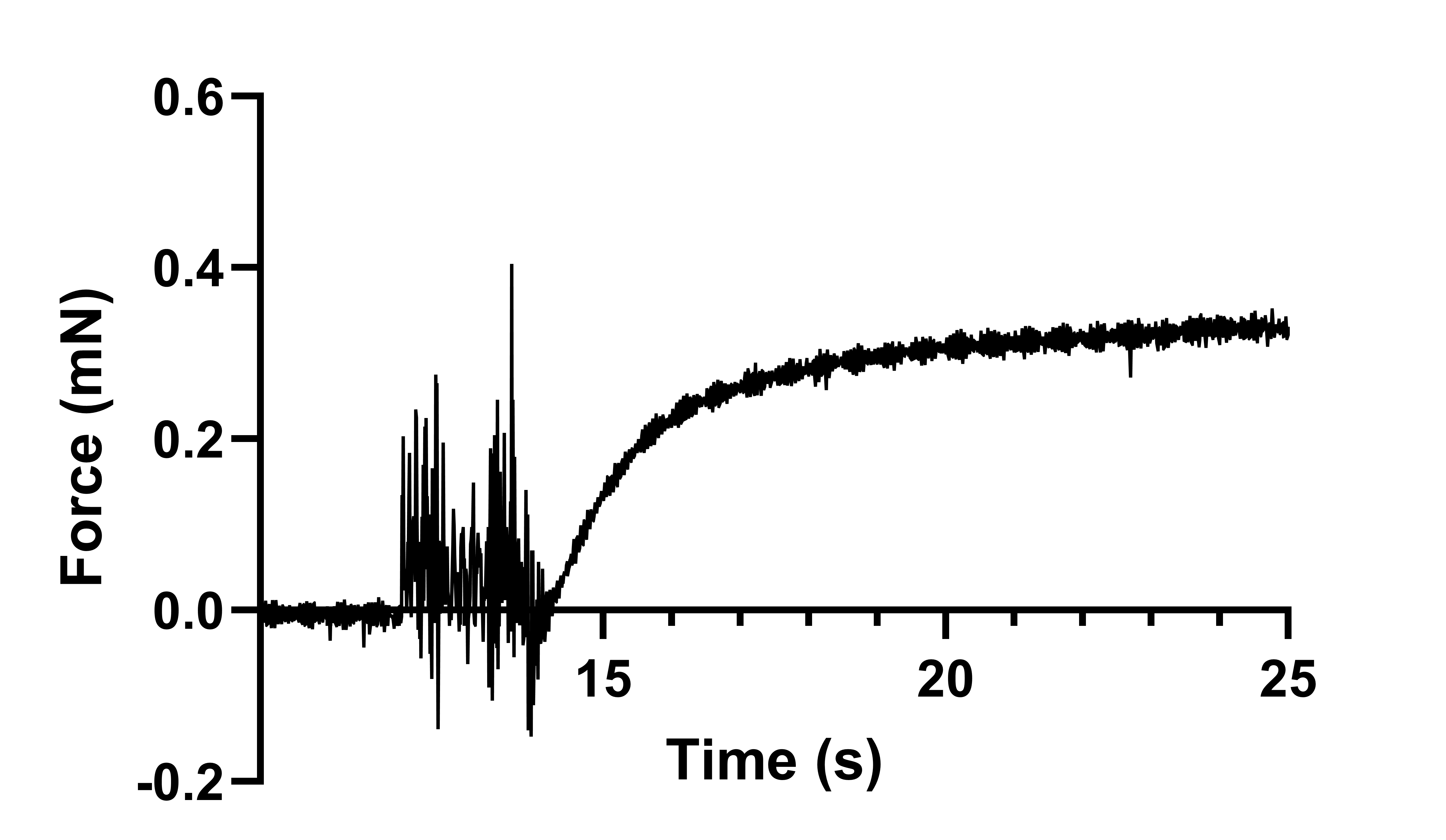

Supplement: Supplementary file 1 — Figure S1. Maximal active force trace at an average sarcomere length (SL) of 2.4 μm (diameter of 0.054 mm). A single skinned muscle fiber was set to SL = 2.4 μm in relaxing solution, transferred through a washing solution, and then to an activating solution (pCa 4.2) to maximally activate the fiber. Sudden changes in the force trace correspond to the testing system transferring between baths. [file PHY2-13-e70645-s003.tif]

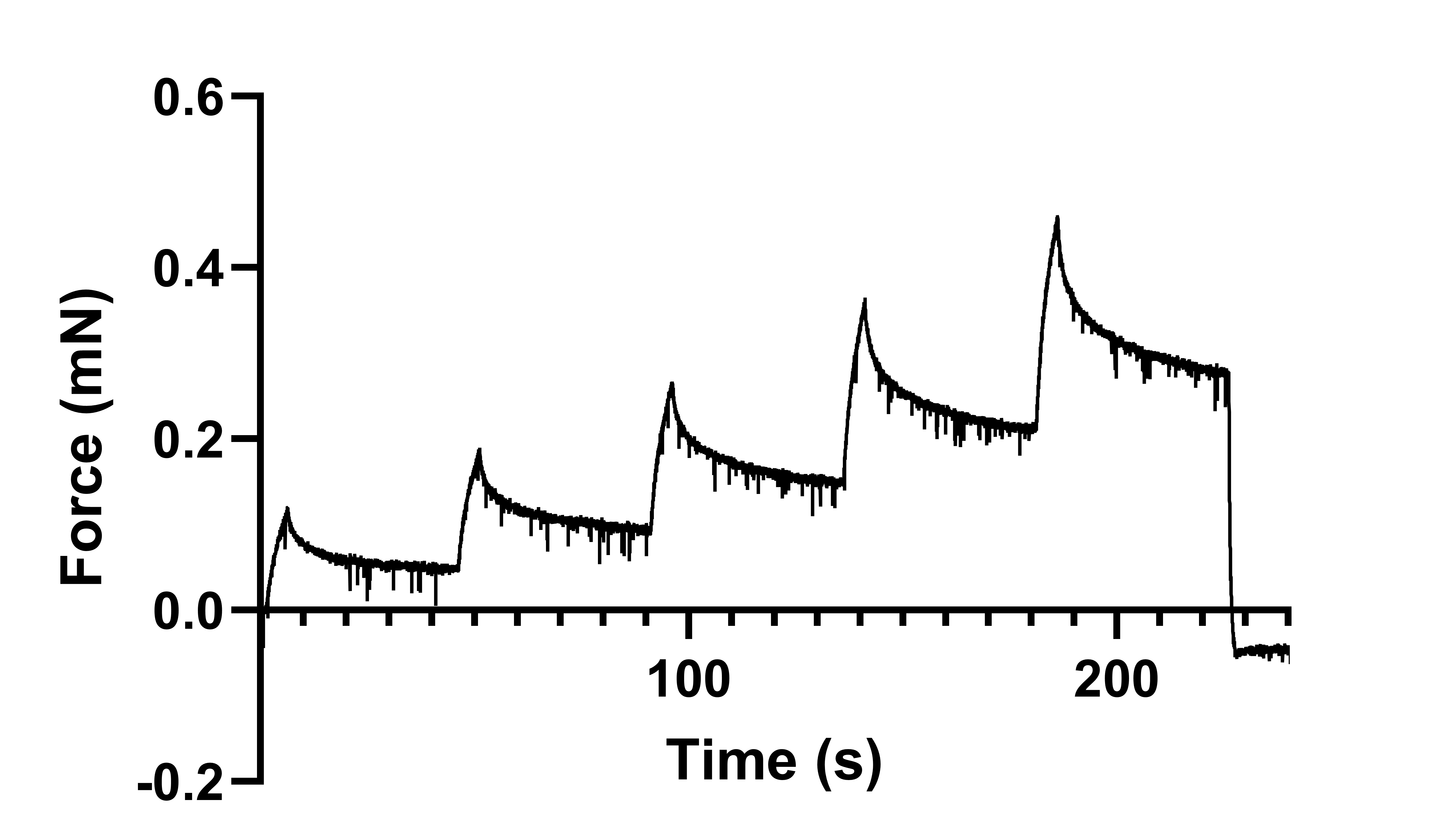

Supplement: Supplementary file 2 — Figure S2. Passive staircase force trace across increasing sarcomere lengths (SL 2.6–3.4 μm, diameter: 0.083 mm). Peak force was collected at the highest point during the stretch, and steady‐state force was collected at the plateau before the next stretch began. [file PHY2-13-e70645-s002.tif]

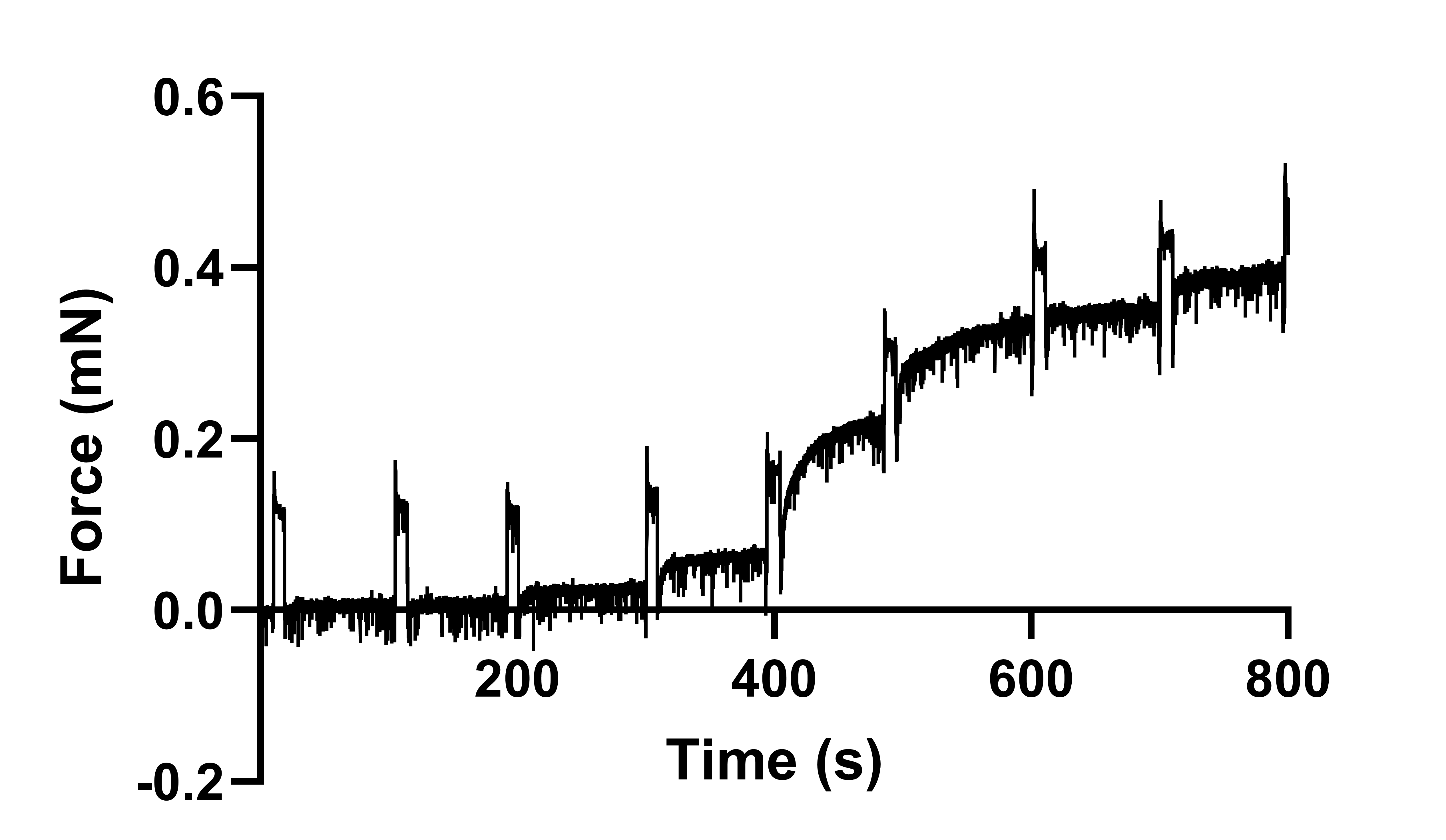

Supplement: Supplementary file 3 — Figure S3. Calcium‐sensitivity force trace at an average sarcomere length (SL) of 2.4 μm with a diameter of 0.033 mm. A single skinned muscle fiber was exposed to solutions of increasing [Ca2+] (pCa 6.8, 6.6, 6.4, 6.2, 6.0, 5.8, 5.6, 5.4). The sudden changes in force are due to the switching of pCa solutions. Baseline force was calculated and subtracted from the active force, which was collected at the plateau before pCa solution change. [file PHY2-13-e70645-s006.tif]

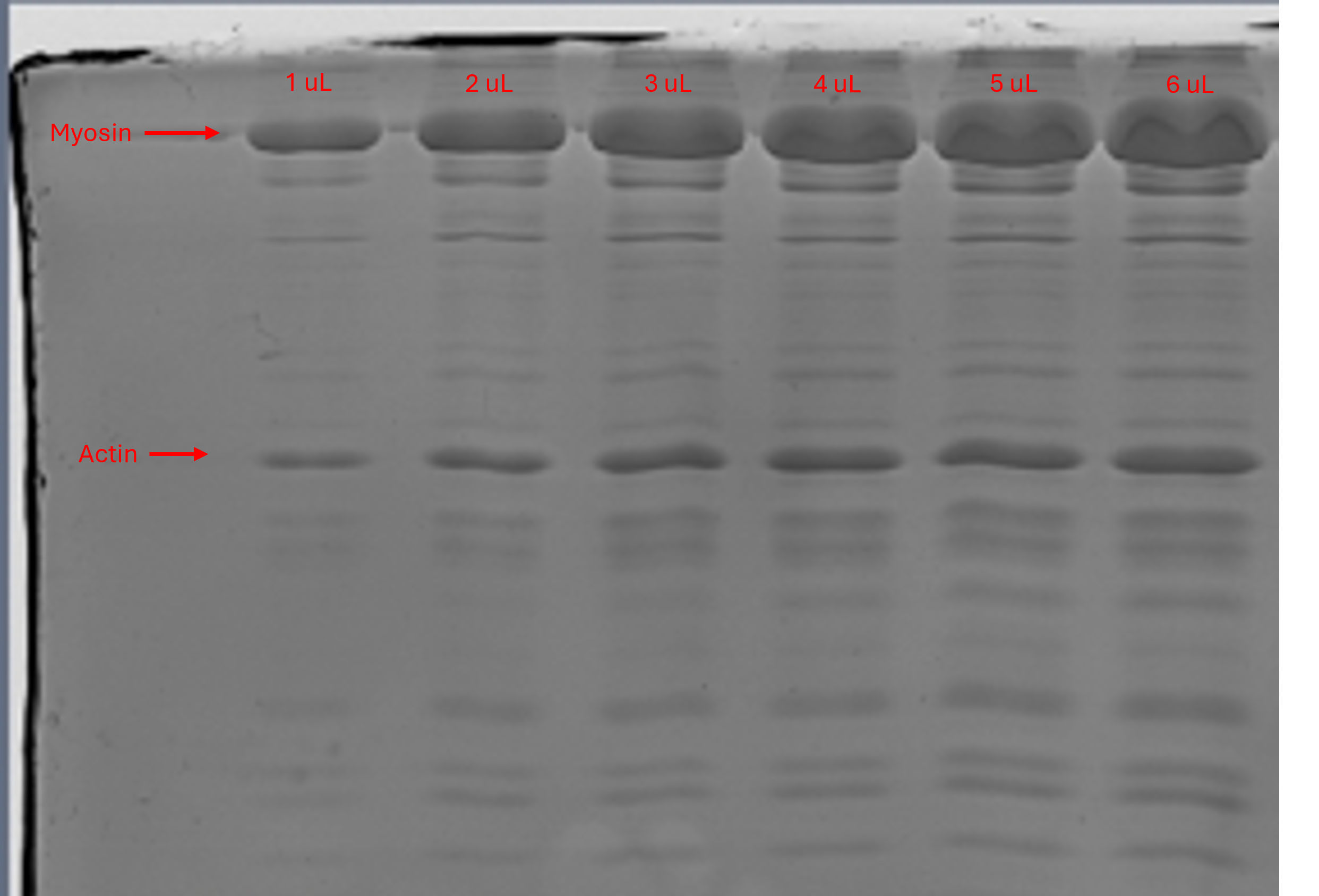

Supplement: Supplementary file 4 — Figure S4. A 12% SDS–PAGE gel was loaded with increasing volumes of the same muscle sample extract to demonstrate the linear range of optical density for densitometry of myosin and actin. [file PHY2-13-e70645-s001.png]

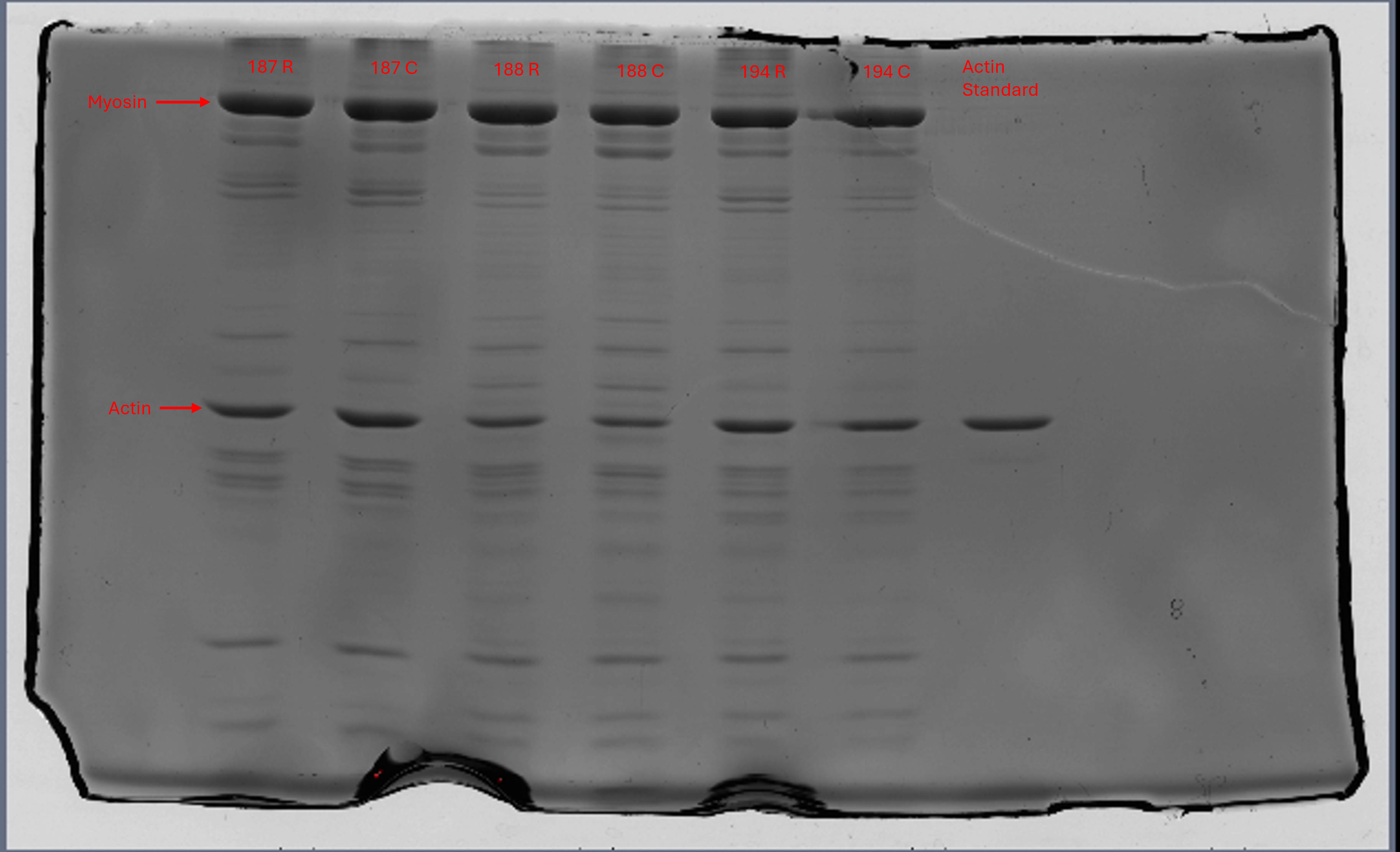

Supplement: Supplementary file 5 — Figure S5. SDS–PAGE gel of collagenase‐incubated samples. The last lane contains a purified actin standard used for band identification. [file PHY2-13-e70645-s005.png]

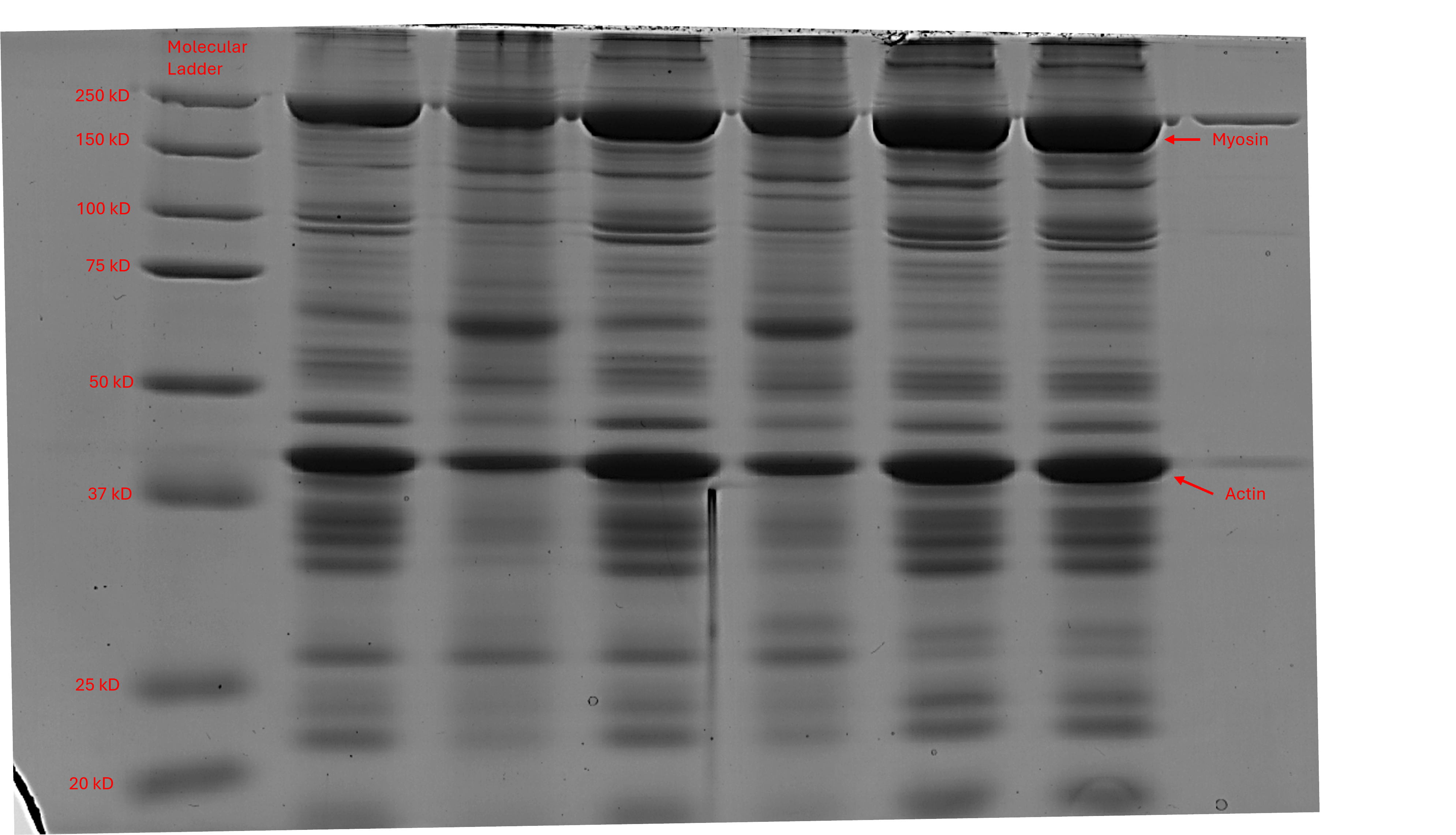

Supplement: Supplementary file 6 — Figure S6. SDS‐PAGE gel of samples run alongside a molecular weight ladder with myosin and actin labeled. Alignment with the ladder was used to verify band identity. [file PHY2-13-e70645-s004.png]
